# Challenges and achievements in the utilization of the health system among adolescents in a region of Burkina Faso particularly affected by poverty

**DOI:** 10.1186/s12913-023-10052-2

**Published:** 2023-10-11

**Authors:** Joshua Krohn, Mamadou Bountogo, Lucienne Ouermi, Ali Sie, Till Baernighausen, Guy Harling

**Affiliations:** 1 Heidelberg Institute of Global Health (HIGH), University of Heidelberg, Im Neuenheimer Feld 130.3, Marsilius-Arkaden, 69120 Heidelberg, Germany; 2 Centre de Recherche en Sante de Nouna, Nouna, Burkina Faso; 3 Africa Health Research Institute (AHRI), Somkhele and Durban, South Africa; 4 Department of Global Health and Population, Harvard T.H. Chan School of Public Health, Boston, MA USA; 5 Institute for Global Health, University College London, London, UK

## Abstract

**Objective:**

Healthcare for adolescents receives little attention in low-income countries globally despite their large population share in these settings, the importance of disease prevention at these ages for later life outcomes and adolescent health needs differing from those of other ages. We therefore examined healthcare need and use among adolescents in rural Burkina Faso to identify reasons for use and gaps in provision and uptake.

**Methods:**

We interviewed 1,644 adolescents aged 12-20 living in rural northwestern Burkina Faso in 2017. Topics included healthcare need and satisfaction with care provided. We calculated response-weighted prevalence of perceived healthcare need and utilization, then conducted multivariable regression to look at predictors of need, realized access and successful utilization based on the Andersen and Aday model.

**Results:**

43.7 [41.2 - 46.0] % of participants perceived need for healthcare at least once in the preceding 12 months - 52.0 [48.1 - 56.0] % of females and 35.6 [32.5 - 39.0] % of males. Of those with perceived need, 92.6 [90.0 - 94.3] % were able to access care and 79.0 [75.6 - 82.0] % obtained successful utilization. Need was most strongly predicted by gender, education and urbanicity, while predictors of successful use included household wealth and female guardian’s educational attainment.

**Conclusion:**

Healthcare utilization among adolescents is low in rural Burkina Faso, but mostly thought of as sufficient with very few individuals reporting need that was not linked to care. Future objective assessment of healthcare need could help identify whether our results reflect a well-functioning system for these adolescents, or one where barriers lead to low awareness of needs or low expectations for service provision.

## Introduction

Adolescence is “a critical phase in life for achieving human potential” [1]; prevention of morbidity and mortality at this age is thus an important investment in the future [2, 3]. Ninety percent of the world’s 1.2 billion adolescents (aged 10 to 19) live in low- and middle-income countries (LMIC) [4–6]. Sub-Saharan Africa (SSA) has the highest proportion of adolescents of any region in the world, and it is the only region of the world in which the number of adolescents continues to rise significantly [4]. An even greater proportion (97 %) of deaths in young people aged 10 to 24 occur in LMIC, with over 60% of all deaths at these ages occurring in Sub-Saharan Africa and southeast Asia [6]. While mortality rates and disability adjusted life years lost have fallen for SSA children over the past 30 years, reductions for adolescents have been less pronounced [5, 7].

The most important causes of ill health in adolescence are very different from those at other life stages, but habits generated during this phase of life are key to determining later-life risk [8]. The burden of adolescent ill-health in Higher Income Countries Higher Income Countries (HIC) is relatively well understood, with injuries, mental health and substance use being the main causes of morbidity and mortality. The burden of adolescent health in LMIC has also been clearly quantified, and differs from that in HIC. Sexual and reproductive health SRH and communicable diseases (especially in SSA) are more important, however, as at other ages, LMIC adolescents are increasingly facing a multiple burden as conditions prevalent in HIC rise too [7, 9].

Healthcare utilization in LMIC at all ages differs from HIC, with significantly higher utilisation rates for outpatient visits and inpatient admissions in HIC [10]. Data on healthcare utilization amongst SSA adolescents is very limited but suggests low coverage of adolescent health concerns including mental health and nutrition [11]. What work has been done continues to be mainly on maternal and child care and sexually transmitted infections (STIs), especially HIV [12–16]. Similarly, key determinants of need for, and utilization of, healthcare are well-studied in HIC, with socioeconomic status, gender and literacy/school enrolment playing important roles, but much less is known for LMIC adolescents aside from their generally low levels of interaction with the healthcare system [17, 18, 9]. As a result, it is unclear whether low healthcare utilization among LMIC adolescents is a function of less actual need, low perceived need for care or barriers to access - including travel distance and time, and services unfriendly to adolescents. Notably, poverty as a key source of healthcare disparity has been noted both between and within SSA countries, but little has been done to unpack whether this disparity is due to differ economic obstacles, underestimated needs, or a failure of individuals or the health system to react to needs as they arise [19–21]. Any understanding of the healthcare needs of LMIC adolescents, and adaptation of programmes which have been successful in HIC, will therefore require adjustment to their greater and differing health requirements [22].

Burkina Faso is both one of the poorest countries in the world, with 44% of people living on less than $1.90 per day and one of the youngest, with a median age of 17 and 24% of citizens aged 10 to 19 [5, 23]. The few data on Burkinabe adolescent health are particularly sparse outside of the SRH topics. Fertility rates are high and median age of first birth is under 20 [14, 21]. Fortunately, the burden of HIV and other STIs is low in Burkina Faso [5]. However, knowledge about STIs is very low even compared to other SSA countries [15]. This could be due to the low burden on the one hand, but also ultimatively to insufficient sexual health education, especially in schools; these factors combine to increase risks for STIs [24]. There is thus likely to be substantial need for healthcare services among rural Burkinabe adolescents.

Healthcare provision in Burkina Faso is limited in terms of physician, hospital bed and Community Health Worker (CHW) numbers [25]. This reflects low health expenditures even by SSA standards with over two-thirds of healthcare payments are made privately, largely out-of-pocket [26]. Adult use of healthcare in rural Burkina Faso is limited by several barriers, including a lack of money, low literacy and high distance to care [27–29]. CHW appear to play an increasingly important role in healthcare provision in rural Burkina Faso, which may overcome some of these barriers [30, 31]. However, evidence on healthcare use by adolescents remains scant and important factors influencing adolescent health and healthcare utilization have not been examined [32].

We therefore analysed a recent population-based survey in Boucle du Mouhoun province in north-western Burkina Faso, a mostly rural region bordering Mali with about 1.9 million inhabitants, 23.7% of whom are young people between 10 and 19 years old [33]. In a country comparison it has a higher rate of illiteracy and less infrastructure [34]. In this analysis, we describe healthcare utilization by rural adolescents, to identify levels of their unmet need for care, and to ascertain predictors of and barriers to healthcare utilization in this population.

## Methods

### Setting and sample

We used data from a study conducted at the Centre de Recherche en Santé de Nouna (CRSN), a Health and Demographic Surveillance System (HDSS) site in Boucle du Mouhoun province, north-western BF [35]. This BF study was part of the Africa Research, Implementation Science and Education (ARISE) Network, a collaboration between nine sub-Saharan African institutions, the Harvard T.H. Chan School of Public Health, and the University of Heidelberg [36]. The CRSN HDSS includes 58 villages and the rural town of Nouna, with a total population of about 107.000 in 2015, of who approximately 22.000 were aged 12 to 19.

The study selected 10 villages purposively sampled to ensure variation in ethnicity, and one sector of Nouna. Within each selected village, a random sample of households was drawn from the most recent available CRSN census and all household members aged 12 to 19 years at the date of sampling were invited to participate ($n=1795$).

CRSN census and all 12 to 19 year old household members household members aged 12 to 19 years at the date of sampling were invited to participate ($n = 1795$).

In the selected Nouna sector, 749 ageeligible adolescents were sampled. The ratio of urban to rural individuals respected the ratio seen in the overall HDSS. All 2544 adolescents in the initial sample were sought for interview; 1644 completed a study interview.

Data was collected in November and December 2017 using tablet computers in private spaces around adolescents’ place of residence. Questions were either asked in French or translated into Dioula or Mooré, the most frequently spoken (but rarely written) local languages by fieldworkers. Fieldworker training included translation practice. Sampled individuals had to have been primarily resident in the Nouna HDSS for at least six months to be eligible to participate.

### Measures

The study collected self-reported information on socio-demographics, behaviours, health practices and health outcomes using a questionnaire that was largely derived from the Global School-Based Student Health Survey with some additional questions [37]. We selected measures for this study based on the phase 2 Anderson and Aday model [38].

### Outcome

Respondents were asked whether and how often, in the past 12 months, they had: 1) been admitted to hospital; 2) visited a primary care clinic; 3) visited a traditional healer; or 4) needed these services but been unable to use them. For each service type used they were asked to specify their satisfaction on a fivepoint Likert scale (very dissatisfied, dissatisfied, neutral, satisfied, very satisfied). Following Anderson and Aday, we first evaluated whether respondents needed care or not, and if so whether they were able to access care (realized access) and then were fully satisfied with their care (effective access). For service use we divided respondents into those with: (i) no need (no to all four questions); (ii) fully met need (yes to any service, no to unmet need); (iii) partially met need (yes to any service, yes to unmet need); and (iv) entirely unmet need (no to all services, yes to unmet need). For satisfaction, we divided respondents into those with: a) fully satisfied (only satisfied/very satisfied responses); b) not fully satisfied (any other response); c) no service use. We finally placed respondents into three categories: those with effective access (ii and a), those without effective access (ii and b, iii or iv) and those with no need for access (i).

### Exposures

To be able to measure inequalities and influencing factors for effective access to healthcare we considered predisposing and enabling factors. Relatively immutable, predisposing variables we included were sex (male or female), age, (12 to 13, 14 to 15, 16 to 17 and 18 to 20), highest attained education (none, primary only, post-primary or higher, Muslim school without grade structure), religion (Animist, Catholic, Muslim, Protestant) and ethnicity (Bwaba, Dafin, Mossi, Peulh, Samo, other). We also included enabling factors that may be amenable to activation and therefore important starting points for interventions. These included household asset index, ranked into quintiles within the HDSS, maternal education (none, any) and urbanicity (small town of Nouna, any village) [39].

### Analytic approach

We first described outcome and exposure variables using proportions, testing for differences in each exposure variable by sex in unweighted data, and by level of healthcare need using inverse non-response weights accounting for different participation rates by age, religion, ethnicity and urbanicity. We then created contingency tables and ran chisquared tests of association for survey data using R version 4.3.0 [40]. We then conducted bivariate and multivariable logistic regression to identify factors associated with first need for healthcare among all respondents, and second effective access to healthcare among those reporting any need. Confidence intervals are between 2.5- 97.5 %. For each set of models we first included only predisposing factors, then added enabling ones.

## Results

### Characteristics of respondents

We were able to locate 1644 of 2544 sampled individuals (64.6 %). Ten participants were dropped because of inconsistent answers regarding need and healthcare utilization. Of the remaining 1634 respondents, 58% were male. Half of participants were attending school at the time of interview but two thirds (66.4 %) of respondents’ highest school level was none or primary. Only 12.5% of the female guardians reported any kind of school education. Most participants were Muslims (69.2 %), 27.6% either Catholics or Protestants (Table 1). Females were less likely not to be in school (45.3% vs 53.5 %) and had higher grade attainment, but most other factors were balanced by sex.

**Table 1:** Baseline characteristics of the study population

	Male	Female	Test of difference by sex
	$n = 948$	$n = 686$	
Age			$\tilde{\chi }^2$ = 2.89, $p= 0.41$
12-13	30.5%	33.1%	
14-15	25.1%	26.7%	
16-17	23.1%	20.7%	
18-20	21.3%	19.5%	
Household index			$\tilde{\chi }^2$ = 12.20, $p = 0.02$
Lowest quintile	22.7%	16.2%	
Second quintile	19.3%	21.4%	
Third quintile	21.8%	21.1%	
Fourth quintile	17.5%	19.4%	
Highest quintile	18.7%	21.9%	
Level of education female Guardian			$\tilde{\chi }^2$ = 2.23, $p = 0.14$
no school education	88.6%	86.0%	
Urbanicity:			$\tilde{\chi }^2$ = 15.22, $p<$ 0.001
urban	26.1%	35.1%	
School status			$\tilde{\chi }^2$ = 12.00, $p = 0.002$
not currently in school	53.5%	45.0%	
in muslim school	0.9%	1.6%	
in regular school	45.6%	53.4%	
Level of education			$\tilde{\chi }^2$ = 7.49, $p= 0.02$
no school education or school without degree	26.8%	24.3%	
low school education	42.1%	38.0%	
higher school education	31.1%	37.6%	
Religion			$\tilde{\chi }^2$ = 0.72,$p = 0.87$
Muslim	69.5%	69.1%	
Catholic	20%	21.1%	
Protestant	7.3%	6.4%	
Animist	3.2%	3.4%	
Ethnicity			$\tilde{\chi }^2$ = 16.34, $p = 0.006$
Peulh	10.1%	10.2%	
Bwaba	19.4%	20.0%	
Dafin	39.8%	36.0%	
Mossi	15.2%	21.0%	
Samo	14.1%	10.3%	
other	1.4%	2.5%	

### Main outcomes

**Fig. 1:**
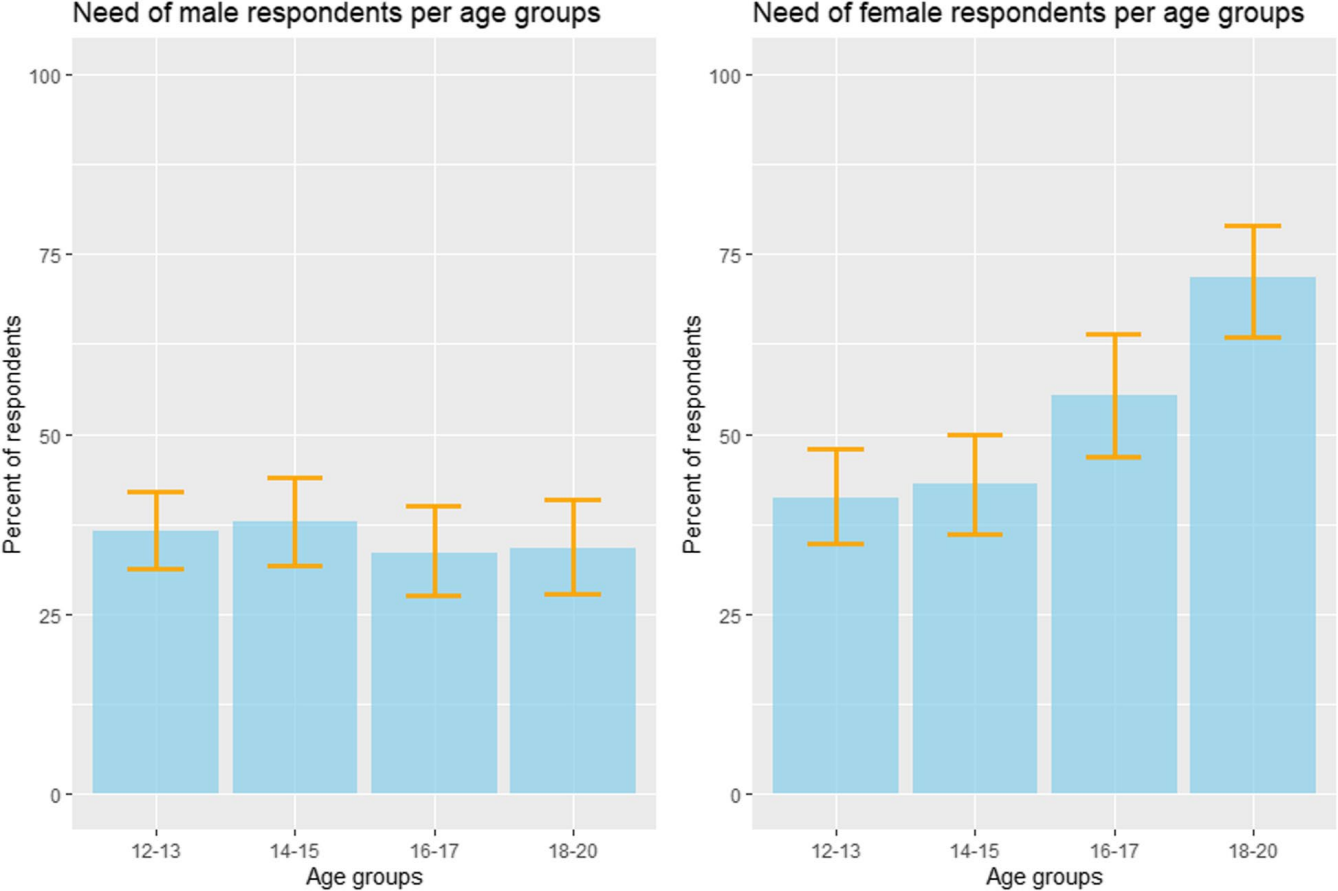
Need of respondents by age groups in percent

Using weighted percentages, less than half of participants (43.7% [Confidence Intervall [CI] 41.2 - 46.0 %]) stated that they had a need for healthcare within the past 12 months (Table 2). This value was consistent with age for males and females up to age 15, but then rose rapidly to 72% for 18-20 year-old women (Fig. 1). While only 39.7% of participants in the lowest quintile stated need, amongst the wealthiest it was 52.1% ($p = 0.009$).

**Table 2:** Different kinds of perceived need according to Andersen and Aday in percentages

Kinds of need:	None	Met	Partially met	Unmet	X-squared and *p*-value
	56.3%	37.3%	3.2%	3.3%	
Gender					$\tilde{\chi }^2$ = 45, *p*-value $< 0.001$
female	48.0%	44.3%	4.0%	3.7%	
male	64.5%	30.4%	2.3%	2.8%	
Age					X-squared = 22, $p = 0.02$
12-13	61.3%	33.0%	2.5%	3.3%	
14-15	59.5%	35.4%	2.4%	3.7%	
16-17	55.2%	38.2%	2.8%	3.8%	
18-20	48.0%	43.3%	5.3%	3.5%	
Household index					$\tilde{\chi }^2$ = 29, $p= 0.01$
Lowest quintile	60.3%	32.5%	2.8%	4.4%	
Second quintile	60.8%	32.5%	2.4%	4.3%	
Third quintile	55.4%	40.1%	1.4%	3.1%	
Fourth quintile	57.6%	35.9%	4.0%	2.6%	
Highest quintile	47.9%	45.2%	5.2%	1.8%	
Schooleducation of female Guardian:					$\tilde{\chi }^2$ = 3, $p= 0.40$
no school education	56.8%	36.6%	3.3%	3.3%	
any kind of school education	53.0%	42.1%	2.0%	3.0%	
Urbanicity:					$\tilde{\chi }^2$ = 27, $p<$ 0.001
rural	60.0%	35.0%	2.5%	2.5%	
urban	47.6%	42.7%	4.9%	4.9%	
School status					$\tilde{\chi }^2$ = 23, $p = 0.002$
not currently in school	60.9%	33.6%	3.1%	2.4%	
in muslim school	52.9%	35.3%	11.8%	0.0%	
in regular school	51.6%	33.6%	3.1%	2.4%	
Level of education					$\tilde{\chi }^2$ = 28, $p<$ 0.001
no school education or school without degree	63.9%	30.7%	2.8%	2.6%	
low school education	58.3%	35.9%	3.5%	2.4%	
higher school education	48.5%	43.7%	3.1%	4.7%	
Religion					$\tilde{\chi }^2$= 18, p = 0.05
Muslim	59.0%	34.6%	3.4%	3.0%	
Catholic	49.7%	44.1%	2.4%	3.9%	
Protestant	52.2%	39.1%	5.2%	3.5%	
Animist	47.1%	49.0%	0.0%	3.9%	
Ethnicity					$\tilde{\chi }^2$= 29, *p*-value = 0.05
Peulh	65.4%	30.7%	2.2%	1.7%	
Bwaba	53.1%	39.8%	2.8%	4.4%	
Dafin	58.6%	35.7%	3.5%	2.2%	
Mossi	54.8%	37.3%	3.5%	4.4%	
Samo	49.5%	45.1%	2.7%	2.7%	
other	44.8%	34.5%	6.9%	13.8%	

Adjusted for survey weights, Pearson’s $X^2$: Rao and Scott adjustment

Perceived need varied by both predisposing and enabling factors. With lowest levels amongst males in poorer households (32.0 %/25.5% in 5th/4th quintile, $p = 0.007$), not in school (29.1 %, $p<$ 0.001), or with no school attainment (26.9 %, $p<$0.001). So school education of the participants has an impact but it gets smaller in the multivariate regression indicating that there are correlations between age, school education, urbanicity and wealth (Table 3). Muslims reported a lower need than participants with other religious affiliations (41 %, $p = 0.01$). There was more demand in Nouna town (52.4 %) than in the villages (40.0 %) ($p<$0.001).

**Table 3:** Multivariate analysis of participants with perceived need over the course of 12 months, weighted on individual level

	Percentages	Predisposing factors only	Predisposing and enabling
Predictors	Percent	OR [CI]	OR [CI]
Gender			
male	35.6	Reference	
female	52.0	1.99 [1.62-2.46]	2.02 [1.63-2.49]
Age			
12-13	38.8	Reference	
14-15	40.5	1.05 [0.78-1.42]	1.06 [0.78-1.43]
16-17	44.7	1.27 [0.92-1.76]	1.26 [0.90-1.75]
18-20	52.0	1.96 [1.40-2.74]	1.84 [1.31-2.59]
Level of education			
no school education or			
school without degree	36.1	Reference	
low school education	41.7	1.11 [0.81-1.52]	1.03 [0.74-1.43]
higher school education	51.5	1.29 [0.85-1.95]	1.07 [0.69-1.43]
School status			
in muslim school	50.0	Reference	
in regular school	48.4	0.81 [0.33-2.01]	1.05 [0.41-2.69]
not currently at school	39.1	0.62 [0.25-1.54]	0.82 [0.32-2.12]
Ethnicity			
Peulh	34.6	Reference	
Bwaba	47.1	1.08 [0.63-1.86]	1.16 [0.67-2.01]
Dafin	41.4	1.23 [0.83-1.81]	1.25 [0.84-1.86]
Mossi	45.3	1.38 [0.90-2.13]	1.35 [0.87-2.10]
Samo	50.5	1.67 [1.05-2.65]	1.53 [0.96-2.46]
other	53.6	1.94 [0.77-4.87]	1.43 [0.56-3.64]
Religion			
Muslim	41.0	Reference	
Catholic	50.3	1.49 [1.02-2.17]	1.69 [1.15-2.49]
Protestant	47.4	1.38 [0.83-2.29]	1.64 [0.98-2.74]
Animist	52.9	1.71 [0.91-3.20]	1.98 [1.06-3.72]
Household index			
Lowest quintile	39.7		Reference
Second quintile	39.2		0.89 [0.63-1.24]
Third quintile	44.6		1.12 [0.80-1.58]
Fourth quintile	42.6		0.99 [0.69-1.42]
Highest quintile	52.1		1.23 [0.84-1.80]
Schooleducation of female Guardian:			
any kind of school education	47.0		Reference
no school education	43.2		1.14 [0.81-1.60]
Urbanicity:			
rural	40.0		Reference
urban	52.4		1.53 [1.15-2.04]
Observations		1634	1634

*CI* Confidence interval, *OR* Odds ratio

One-third of respondents (37.3 %) reported that they had always been able to use a healthcare provider - over 85% of all those reporting any need. Primary care centers were used most frequently as health care providers by the adolescents (33.5% [CI 31.2 - 36.0 %]), to a lesser extent traditional healers (9.3% [CI 7.9 - 11.0 %]) and the Nouna hospital (7.4% [CI 6.1 - 9.0 %]. Only a small minority of respondents reported partially (3.2 %) or entirely (3.3 %) unmet need for healthcare. The proportion of participants with entirely unmet need was similar amongst the participant characteristics, although the figure was lower for those from richer households and rural areas (Table 2).

**Fig. 2:**
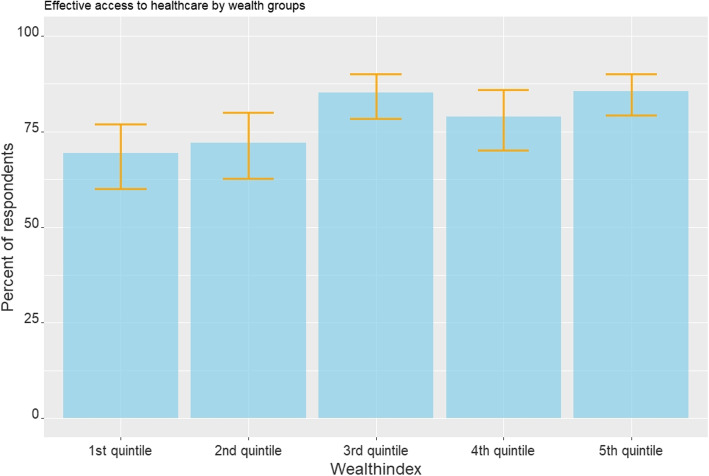
Impact of wealthindex on efficient access to healthcare

Almost four in five participants (79 %) reported effective access to healthcare. In contrast to the stated need, where predisposing factors played an important role, enabling factors particularly influenced effective / successful access to healthcare, except that Bwaba (Odds ratio [OR] 0.23 [ CI 0.08 - 0.71]) and other minority ethnic groups (OR 0.16 [CI 0.04 - 0.66]) had poorer access than members of other ethnic groups. With increasing prosperity, the healthcare of the participants was better (Fig. 2), with the highest quintile having 2.97 times the odds (CI 1.51-5.95) of receiving efficient access compared to the lowest quintile. The participants whose mother had no school education also received worse care (OR 0.43 [CI 0.20-0.84]) (Table 4). Accounting for those with no need and those without access to care overall 59.5% of all participants never had any kind of contact with the healthcare system mostly because they had no perceived need (56.3 %). Thus, of those in need, 92.6% [CI 90.0 - 94.3 %] had access to the healthcare system.

**Table 4:** Multivariate analysis of successful access to healthcare among those with need

	Predisposing factors only	Predisposing and enabling
Predictors	OR [CI]	OR [CI]
Gender		
male	Reference	
female	0.91 [0.61-1.35]	0.89 [0.60- 1.33]
Age		
12-13	Reference	
14-15	1.63 [0.89-2.98]	1.64 [0.88-3.05]
16-17	1.15 [0.61-2.16]	1.16 [0.58-2.30]
18-20	0.97 [0.51-1.83]	0.94 [0.50-1.78]
Level of education		
no school education or		
school without degree	Reference	
low school education	1.74 [0.91-3.34]	1.74 [0.89-3.41]
higher school education	1.46 [0.66-3.27]	1.48 [0.63-3.45]
School status		
in muslim school	Reference	
in regular school	1.46 [0.28-7.55]	1.29 [0.22-7.37]
not currently in school	1.41 [0.27-7.34]	1.30 [0.23-7.48]
Ethnicity		
Peulh	Reference	
Bwaba	0.24 [0.08-0.72]	0.23 [0.08-0.71]
Dafin	1.05 [0.45-2.43]	0.89 [0.37-2.12]
Mossi	0.69 [0.29-1.66]	0.53 [0.21-1.32]
Samo	1.06 [0.40-2.78]	0.86 [0.32-2.32]
other	0.28 [0.08-1.07]	0.16 [0.04-0.66]
Religion		
Muslim	Reference	
Catholic	2.11 [1.03-4.33]	2.06 [0.95-4.49]
Protestant	1.56 [0.66-3.68]	1.33 [0.53-3.36]
Animist	3.55 [1.09-11.52]	4.15 [1.17-14.68]
Household index		
Lowest quintile		Reference
Second quintile		1.15 [0.62-2.11]
Third quintile		2.61 [1.34-5.09]
Fourth quintile		1.76 [0.86-3.59]
Highest quintile		2.97 [1.48-5.94]
Schooleducation of female Guardian:		
any kind of school education		Reference
no school education		0.41 [0.20-0.85]
Urbanicity:		
rural		Reference
urban		0.67 [0.39-1.14]
Observations	677	677

*CI* Confidence interval, *OR* Odds ratio

### Discussion

Evidence of the perceived need for, and use of, healthcare by adolescents in LMICs in general, and sub-Saharan Africa in particular, is sparse. This is despite their demands on the health system being substantial and different from those of other age groups. It is important to understand not only what adolescent health needs there are and what other factors are important to properly understand their behaviour [38, 41]. We also need to know whether healthcare systems and policy makers are prepared to meet adolescent health demands in order to be able to take the right approaches [42]. The success of these implementations seems to be achievable only when considering all intervention types [43]. We therefore analysed self-reported data on healthcare system need and use by young people in rural north-western Burkina Faso, a region particularly affected by poverty in a country that is already one of the poorest in the world and therefore represents an extreme in a global comparison. More than 40% of participants stating a perceived need for healthcare in the past year, with notably more need among older female adolescents. Still, healthcare need was considerably lower than that seen in HICs [44, 45].

Perceived need was most strongly associated with predisposing demographic factors such as age, gender, education and religion - although it was also higher in urban areas (Table 5). These findings highlight that inequity in perceived healthcare need arises when it is influenced not only by demographic factors and symptom severity, but also by, e.g., the ability to recognize these symptoms and to have the necessary knowledge about the health system and the possibilities of use [46]. This pattern of mainly predisposing factors predicting perceived need independently of enabling factors is similar to that seen in studies of Canadian and US adolescents [41, 47].

**Table 5:** Percentage of participants with perceived need over the course of 12 months. Percentages weighted on individual level

	Total	Male	Female
	43.7 %	35.6%	52.0%
**Age**			
12-13	38.8%	36.5%	41.2%
14-15	40.5%	37.8%	43.3%
16-17	44.7%	33.3%	55.4%
18-20	52.0%	34.2%	71.9%
**Household index**			
Lowest quintile	39.7%	32.0%	50.0%
Second quintile	39.2%	25.0%	50.6%
Third quintile	44.6%	40.9%	48.5%
Fourth quintile	42.6%	35.9%	48.7%
Highest quintile	52.1%	42.1%	62.0%
**School education of female Guardian**			
no school education	43.2%	39.4%	51.7%
had school education	47.0%	35.0%	53.7%
**Urbanicity**			
urban	52.4%	45.0%	59.7%
rural	40.0%	31.7%	48.6%
**School status**			
in muslim school	50.5%	37.5%	60.0%
in regular school	48.4%	43.2%	53.3%
not currently in school	39.1%	29.1%	50.5%
**Level of education**			
no school education or school without degree	36.1%	26.9%	45.9%
low school education	41.7%	35.4%	48.8%
higher school education	51.5%	42.6%	59.3%
**Religion**			
Muslim	41.0%	32.3%	50.0%
Catholic	50.3%	44.4%	55.1%
Protestant	47.4%	37.3%	58.2%
Animist	52.9%	45.8%	57.7%
**Ethnicity**			
Peulh	34.6%	21.6%	47.8%
Bwaba	47.1%	37.3%	55.6%
Dafin	41.4%	29.3%	53.9%
Mossi	45.3%	49.0%	41.4%
Samo	50.5%	40.7%	64.1%
other	53.6%	58.3%	50.0%

Research on healthcare need and use in LMIC often focuses either on level of access or level of demand and the health system’s capacity to meet this demand. In most studies (including ours) it is the same factors that cause inequality in the health system [48]. In contrast to other studies however we did not only limit ourselves to one of these aspects or subgroup, but took into account age, gender, socio-economic background, education and residence, but also religion, ethnicity and maternal education. Our study shows that perceived need is influenced by age and gender, but also residence, ethnicity, religion and wealth suggesting that it matters where you live and from which ethnic or religious background you come from implying that information on health care and needs are still not evenly supplied to everyone.

Among those Burkinabe adolescents perceiving need, over 90% had access to the health system when needed. More than three quarters of those with need had effective access (always available with a high level of satisfaction). These numbers are high and in contrast to the findings of other studies in Sub-Saharan Africa, which find poor accessibility and lower levels of satisfaction [49, 50]. This difference may well reflect healthcare being sufficient for this age group in this setting. However it may also reflect an underestimation of personal needs, or that the level of care required for satisfaction is particularly low. Since this study did not attempt to capture objective healthcare need, we are not able to directly assess which scenario is more likely. In a four-country study of STIs among male adolescents, Burkinabé reported higher healthcare system utilization and greater trust and satisfaction, but also less knowledge of STIs shows a higher level of utilization and greater trust and satisfaction, but also less knowledge of STIs in Burkina Faso than in Ghana, Malawi and Uganda [42]. Were this pattern also true in our study, it might be the case that adolescents are both able to access good care and may not identify certain needs. Additional research - both quantitatively including objective measures of need and qualitatively through indepth discussion of need and use - would help answer these questions.

Predictors of effective HC use were mainly enabling factors, in line with other studies in both LMIC and HIC [48, 51, 52]. While our data suggest high levels of effective access, it was notable that respondents from lower income houses and rural areas had both lower stated need and a higher unmet need amongst those with need. Our findings are therefore in line with other studies showing the poor do not only understate their needs, they or the healthcare system-on average-also respond inadequately to their needs [21]. This reinforces the importance of considering both barriers to care access and to identifying need. It‘s already been shown that knowledge about adequate care seems to be little, care is available, but quality is poor [51]. Use of preventative services, which are particularly important in this age group, were especially low in other studies. The large influence of urbanicity on healthcare use in Burkina Faso is described before while wealth playing the most important factor in all other countries, suggesting ability to reach care may be the primary factor [20]. Our finding that successful access was most strongly associated with wealth and education in our setting suggests that the distribution of healthcare still favours those who can afford it. Interventions that improve access to the poorest, even in generally poor communities, may generate the greatest benefits while also reducing inequality.

### Strengths and limitations

This analysis has several limitations. First, the underlying dataset was cross-sectional, limiting ability to determine the temporal directions of any associations seen, conflating participants’ age and cohort and making evaluation of intra-individual development impossible. Future work following up this cohort could allow such issues to be examined. Second, the study was limited to one geographic area in rural Burkina Faso. While the study sampled from a local population census and non-response weights were used to minimize selection bias, generalization to the national level or beyond should only be done with caution. However, to the best of our knowledge this is a novel study in its examination of the usage patterns of health services for adolescents in Burkina Faso and more widely across low-income settings and is thus an important initial contribution to the literature. Third, needs and utilization were based on self-report, not objectively measured, e.g., by physical examination or medical history. While self-reports capture perceived need, objective measurement would help evaluate if actual needs were over or underreported, either overall or differentially by key covariates. Fourth, the distance to the nearest primary care center or hospital was not included in the analysis, since distance and difficulty of travel between villages differs by precise route and season. As a result it is possible that some of our findings might reflect confounding by difficulty of reaching care. Although unmet need in this population is very low. Finally, the study did not enquire as to why participants needed healthcare, were unable to access it, and were/were not satisfied. Such insight would be a valuable next step in designing interventions.

### Conclusion

In our analysis of healthcare need and utilization by adolescents in rural Burkina Faso, we find relatively low overall contact rates, but very high levels of successful utilization by those self-identifying need. Perceived need was strongly patterned by age, gender, school education and urbanicity, while effective access to care was linked to household wealth and maternal education. Over half of participants did not have contact with the healthcare system in the past twelve months, considerably more than in HIC, where prevention amongst adolescents is increasingly emphasized. Our results support the findings that low education, low household wealth and rurality decrease healthcare access. Further research can extend our work by assessing objective healthcare need and better understanding the context of healthcare need and use, possibly also through other approaches such as in-depth interviews or ethnographic approaches. Such work would help determine the extent to which our results reflect a well-functioning system for these young people, or one where barriers lead to low awareness of needs or low expectations for service provision.

## Data Availability

Data are not publicly available due to consent not being given by participants for data to be shared openly, and due to the risk of deductive disclosure with sufficient local information given the inclusion of large proportions of age cohorts in the study villages. Anonymised data are available from ARISE study data controllers only following signature of a data use agreement restricting onward transmission. Anyone wishing to replicate the analyses presented, or conduct further collaborative analyses using ARISE (which are welcomed and considered based on a letter of intent), should contact Dr Guy Harling (g.harling@ucl.ac.uk) in the first instance.

## Abbreviations

ARISE: Africa Research, Implementation Science and Education
BF: Burkina Faso
CHW: Community Health Worker
CRSN: Centre de Recherche en Santé de Nouna
HDSS: Health and Demographic Surveillance System
HIC: Higher Income Countries
LMIC: Lower and Middle Income Countries
SRH: Sexual and Reproductive Health
SSA: Sub-Saharan Africa
